# Supplementary material for: What do you do if your relief comes to work intoxicated: An Impaired Provider Scenario
Source: J Educ Teach Emerg Med. 2020 Oct 15;5(4):S1–S29. doi: 10.21980/J8DM0H (PMC10332520; doi:10.21980/J8DM0H)
Supplement: Supplementary file 1 [file jetem-5-4-s1-supp1.pptx]

## Slide 1
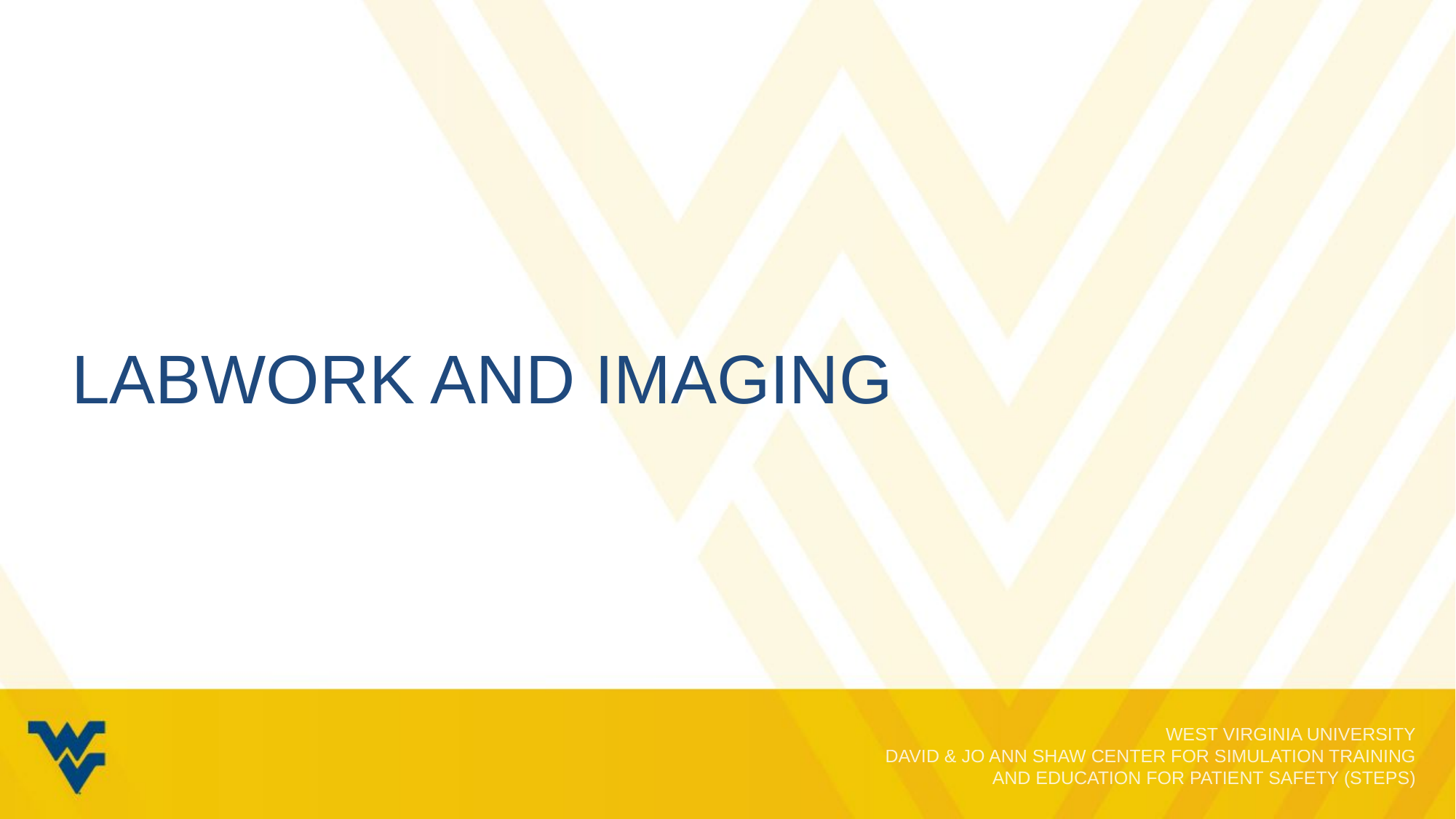

# Labwork and imaging

## Slide 2
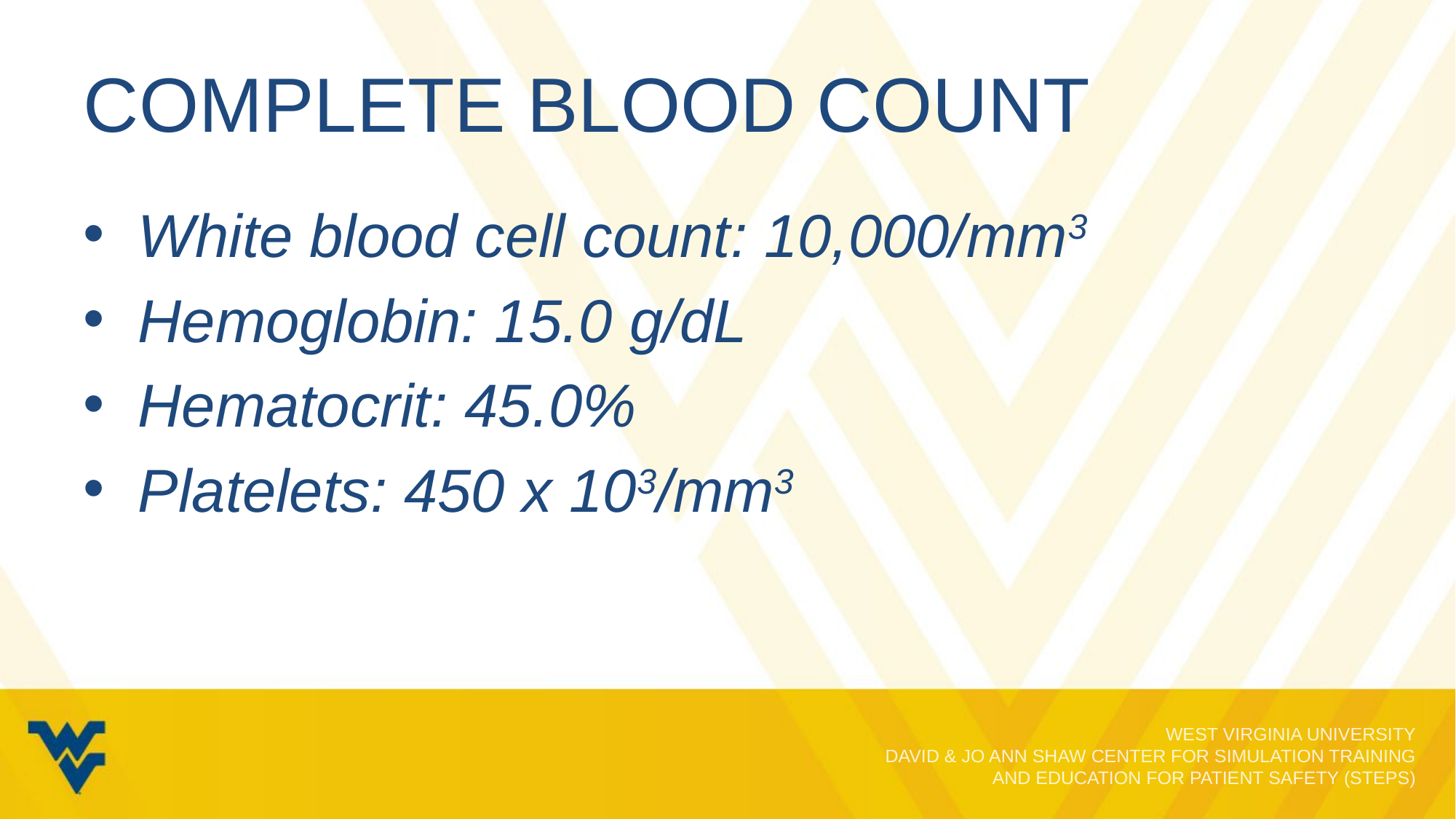

# Complete Blood count
White blood cell count: 10,000/mm3
Hemoglobin: 15.0 g/dL
Hematocrit: 45.0%
Platelets: 450 x 103/mm3

## Slide 3
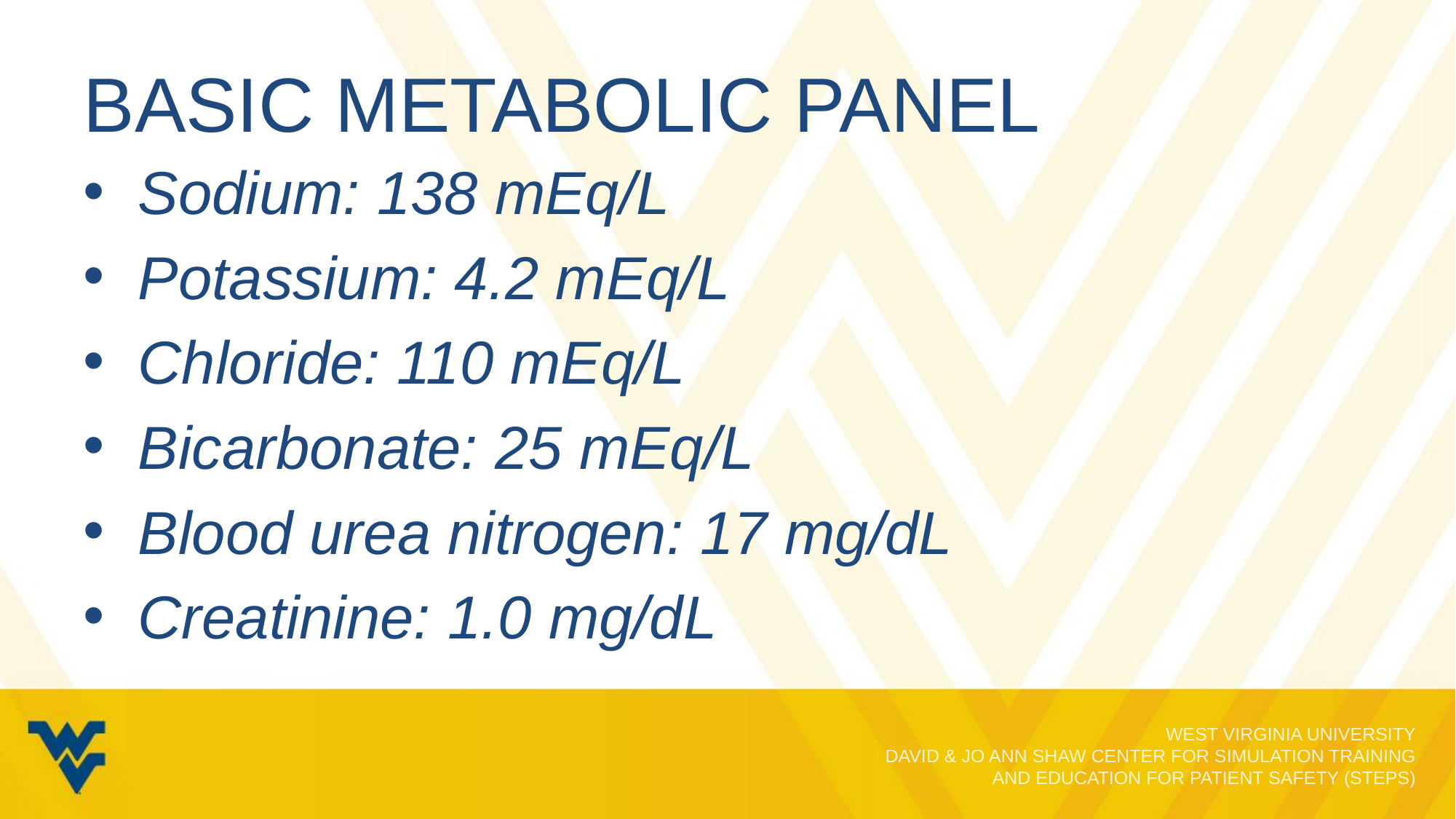

# Basic Metabolic Panel
Sodium: 138 mEq/L
Potassium: 4.2 mEq/L
Chloride: 110 mEq/L
Bicarbonate: 25 mEq/L
Blood urea nitrogen: 17 mg/dL
Creatinine: 1.0 mg/dL

## Slide 4
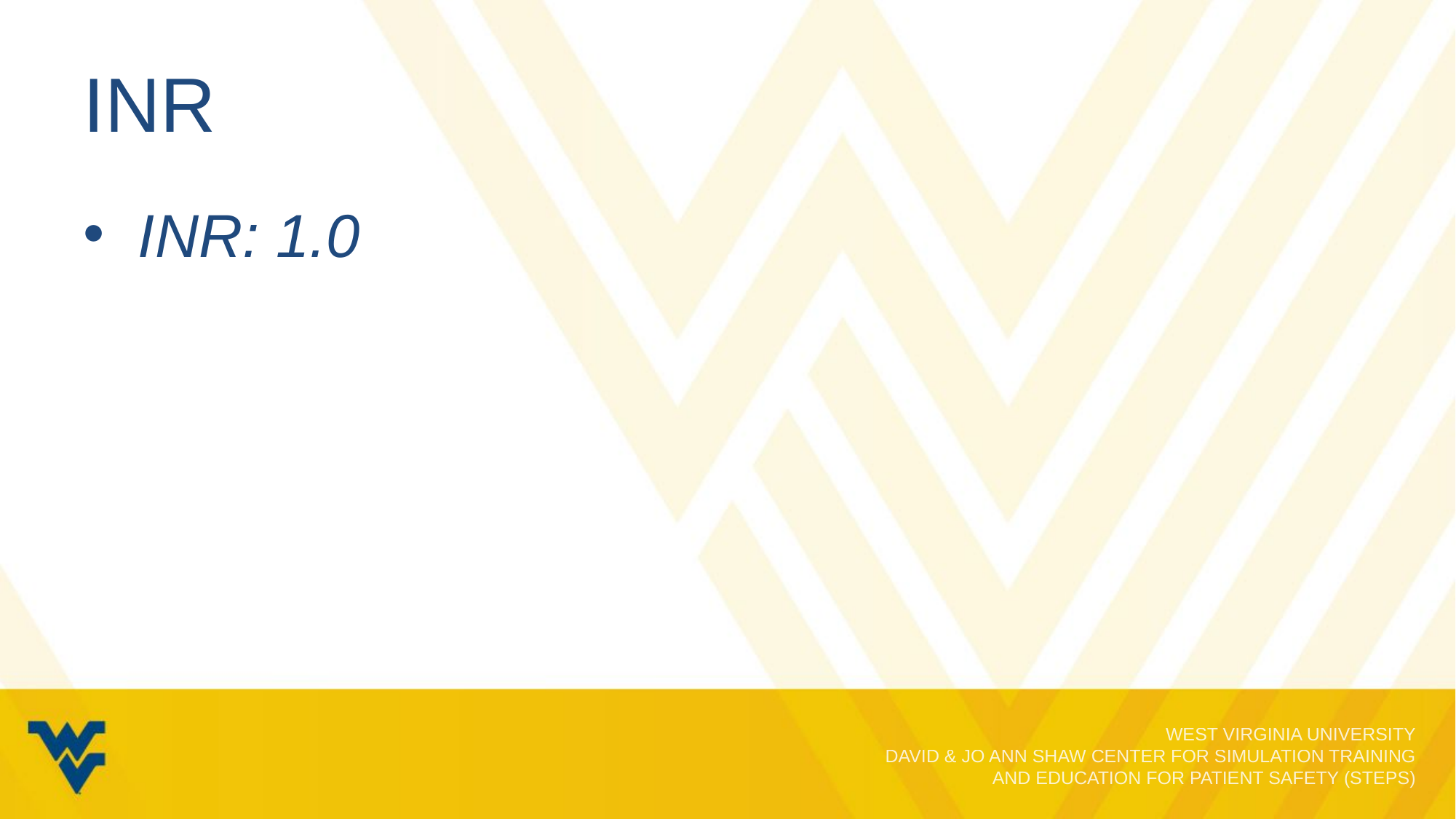

# INR
INR: 1.0

## Slide 5
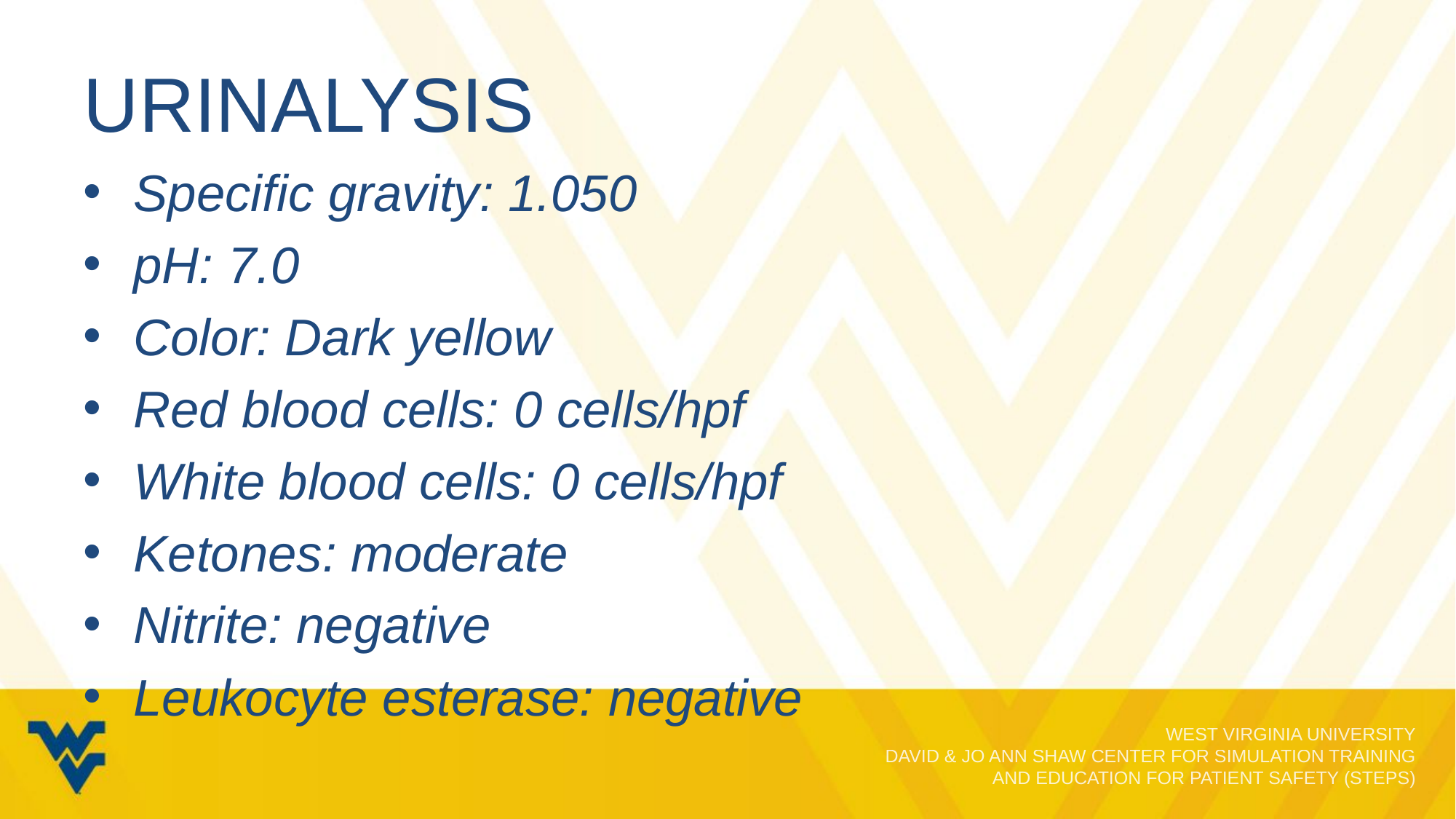

# Urinalysis
Specific gravity: 1.050
pH: 7.0
Color: Dark yellow
Red blood cells: 0 cells/hpf
White blood cells: 0 cells/hpf
Ketones: moderate
Nitrite: negative
Leukocyte esterase: negative

## Slide 6
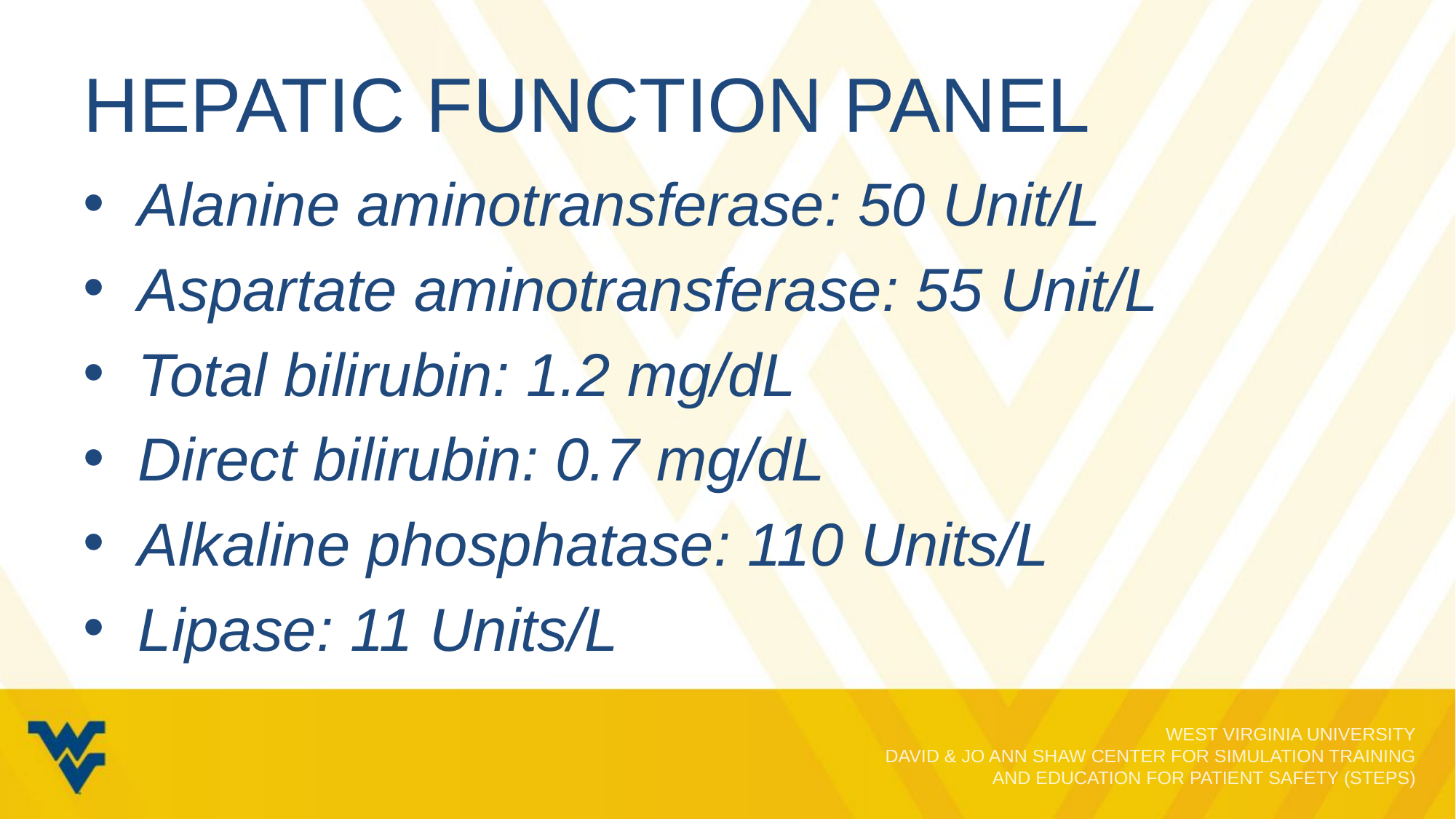

# Hepatic Function Panel
Alanine aminotransferase: 50 Unit/L
Aspartate aminotransferase: 55 Unit/L
Total bilirubin: 1.2 mg/dL
Direct bilirubin: 0.7 mg/dL
Alkaline phosphatase: 110 Units/L
Lipase: 11 Units/L

## Slide 7
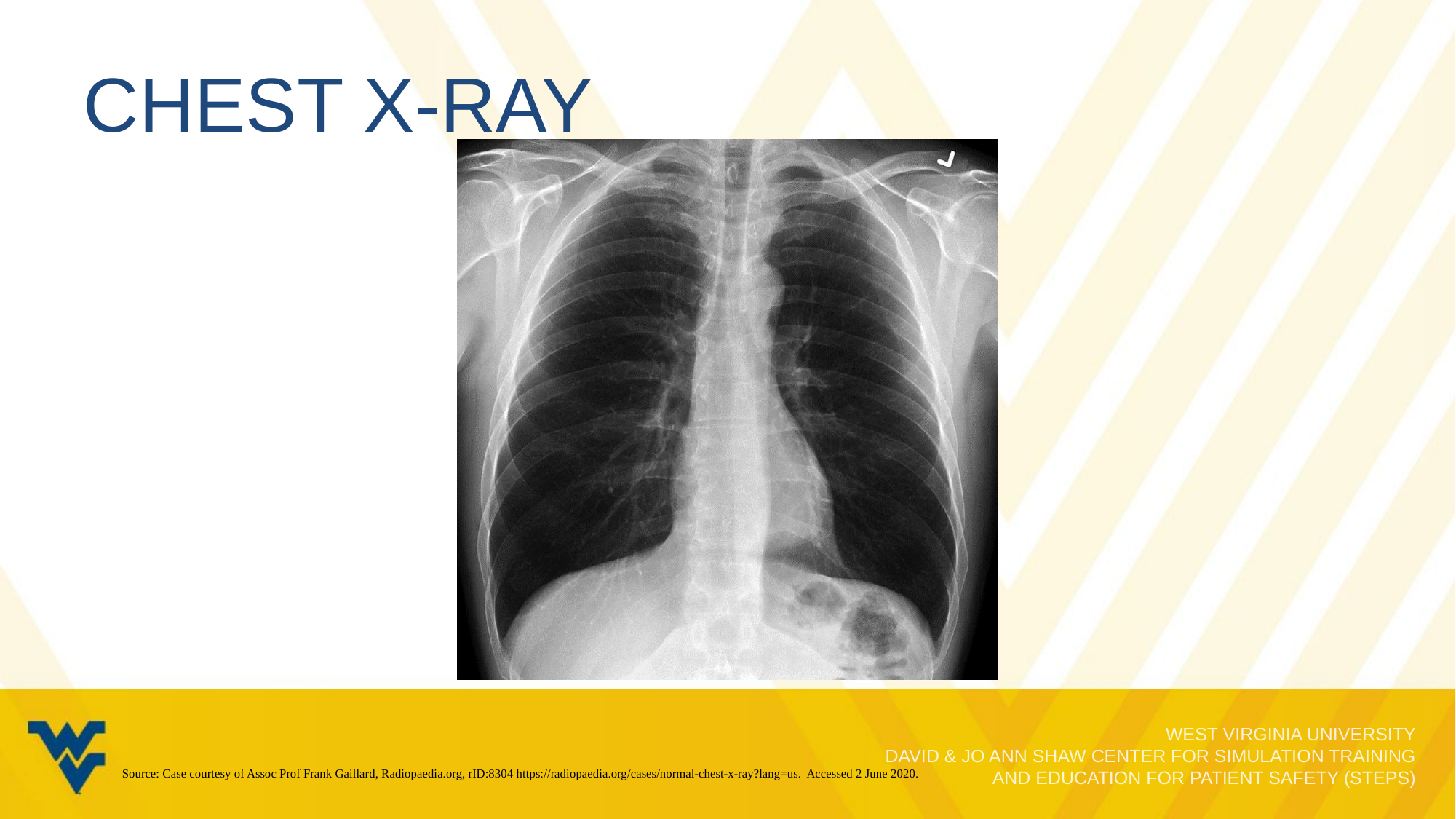

# Chest x-ray
Source: Case courtesy of Assoc Prof Frank Gaillard, Radiopaedia.org, rID:8304 https://radiopaedia.org/cases/normal-chest-x-ray?lang=us. Accessed 2 June 2020.

## Slide 8
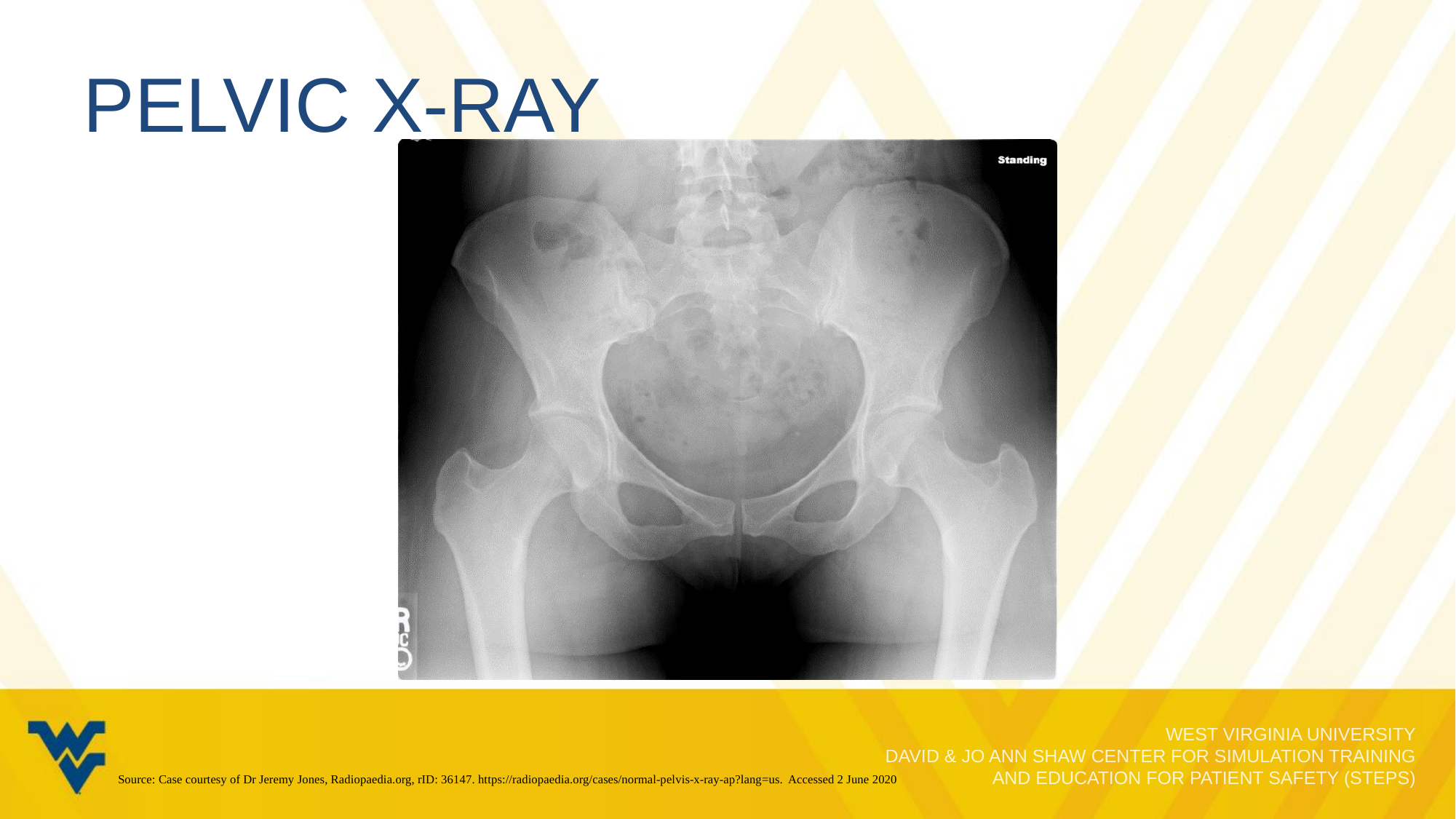

# Pelvic x-ray
Source: Case courtesy of Dr Jeremy Jones, Radiopaedia.org, rID: 36147. https://radiopaedia.org/cases/normal-pelvis-x-ray-ap?lang=us. Accessed 2 June 2020

## Slide 9
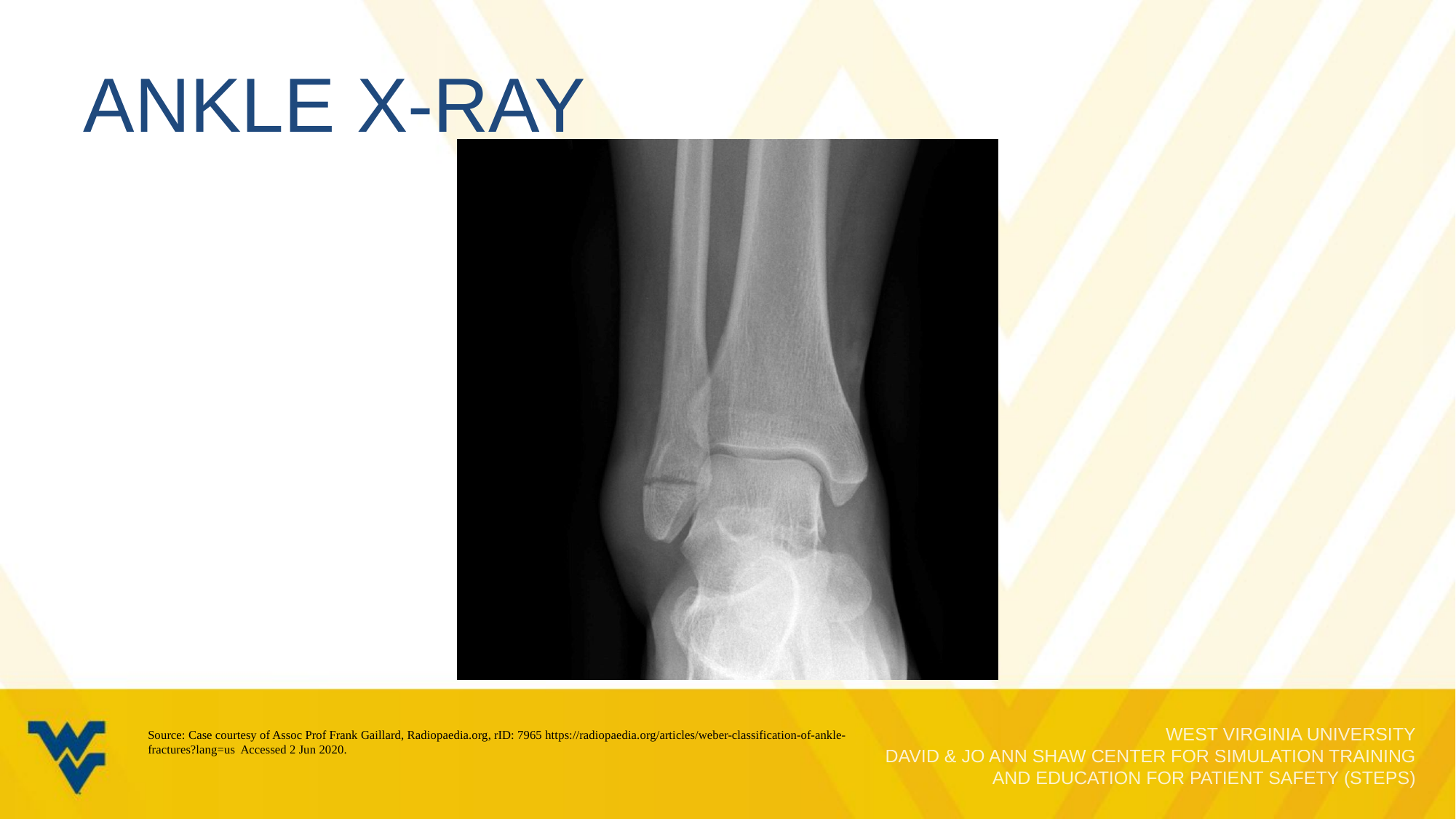

# Ankle x-ray
Source: Case courtesy of Assoc Prof Frank Gaillard, Radiopaedia.org, rID: 7965 https://radiopaedia.org/articles/weber-classification-of-ankle-fractures?lang=us Accessed 2 Jun 2020.

## Slide 10
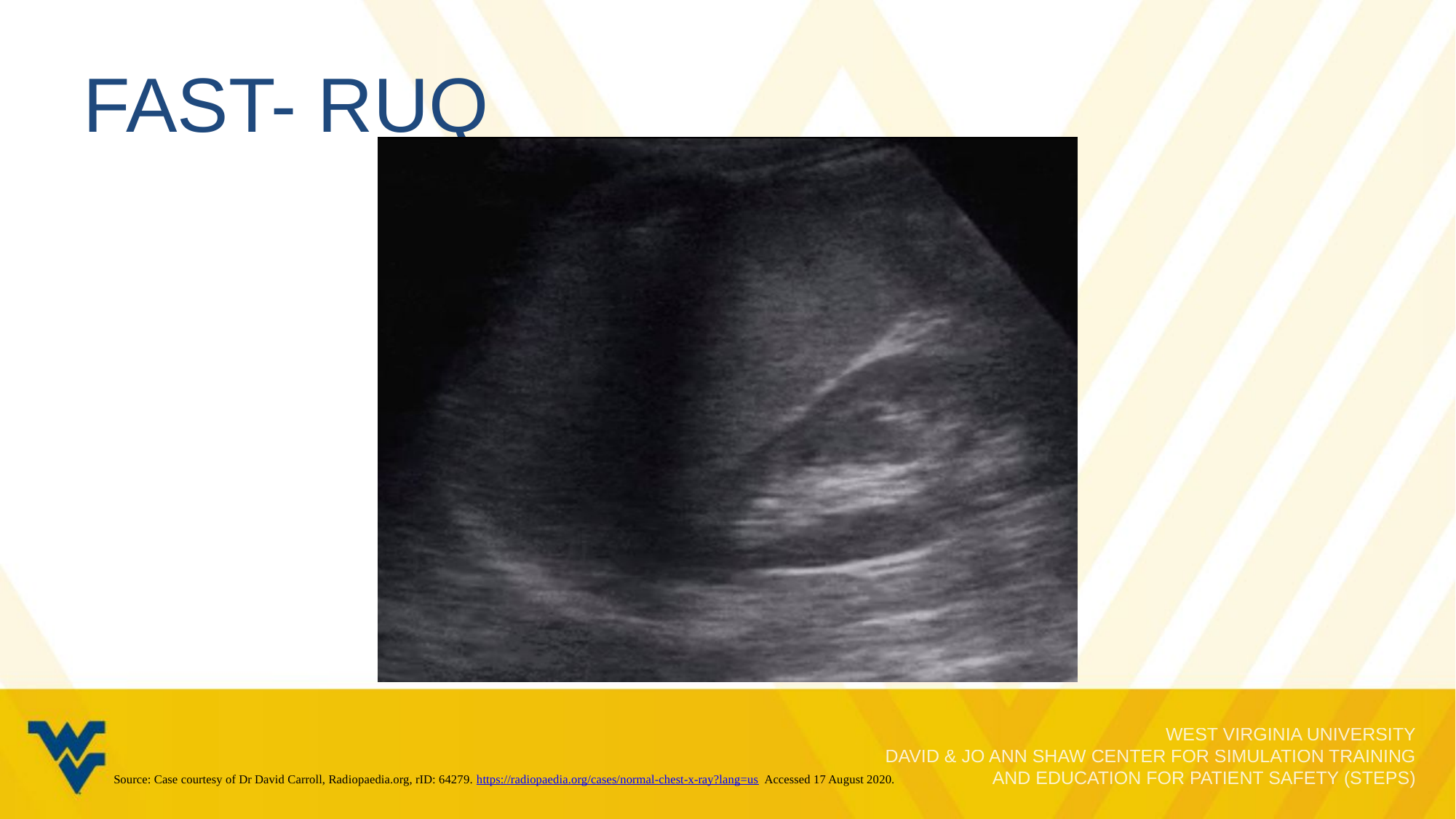

# FAST- RUQ
Source: Case courtesy of Dr David Carroll, Radiopaedia.org, rID: 64279. https://radiopaedia.org/cases/normal-chest-x-ray?lang=us Accessed 17 August 2020.

## Slide 11
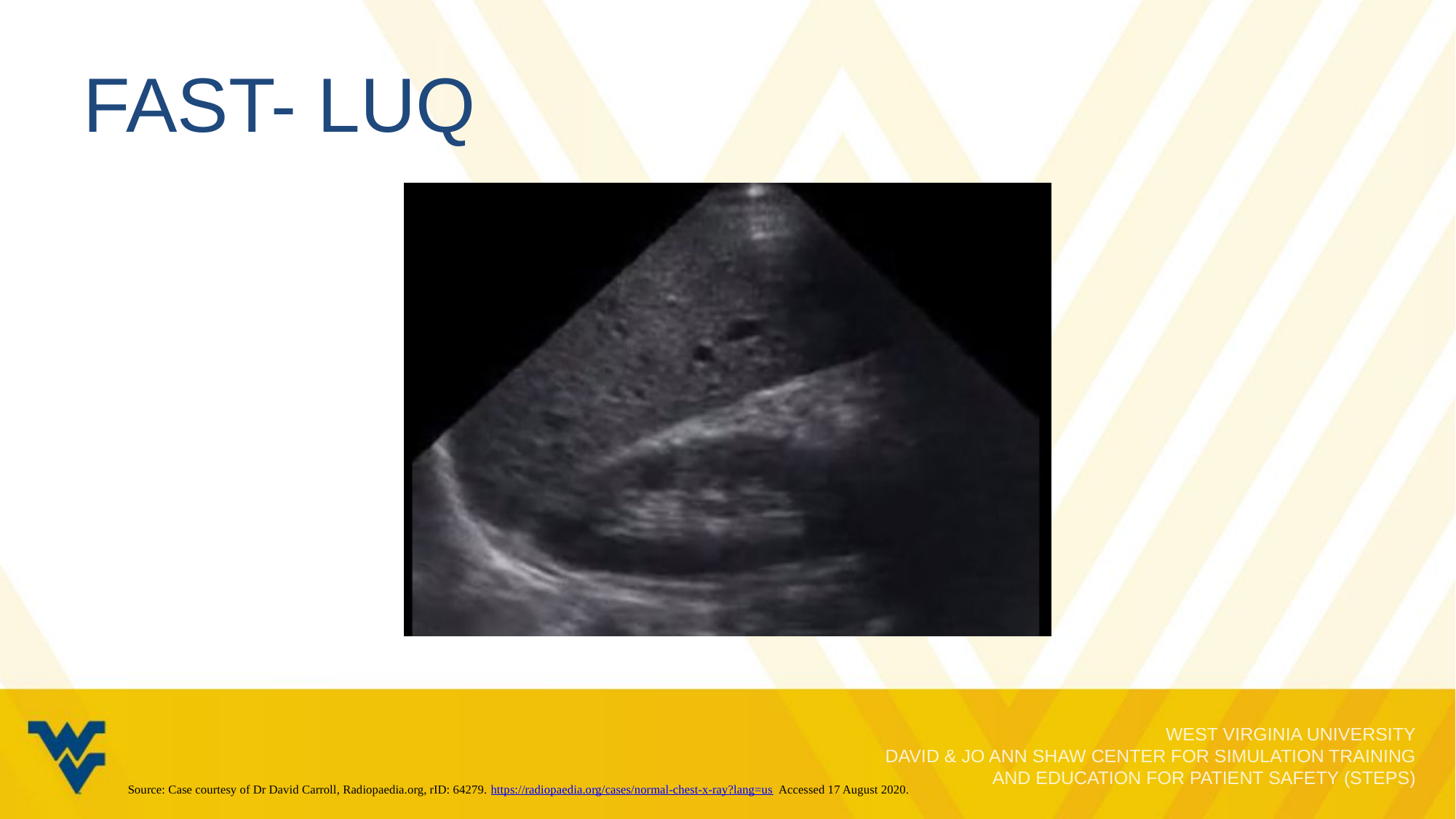

# FAST- luq
Source: Case courtesy of Dr David Carroll, Radiopaedia.org, rID: 64279. https://radiopaedia.org/cases/normal-chest-x-ray?lang=us Accessed 17 August 2020.

## Slide 12
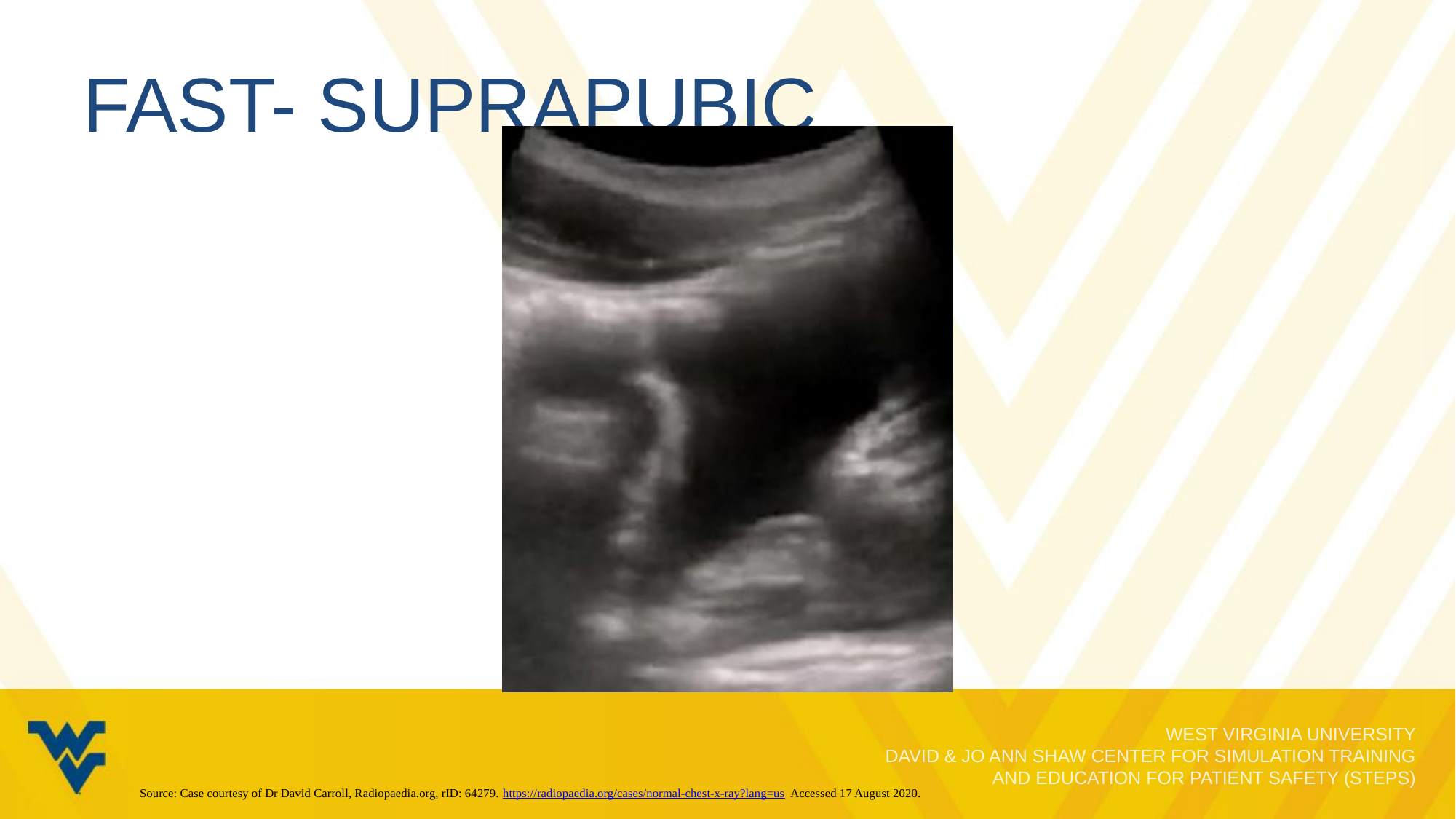

# Fast- suprapubic
Source: Case courtesy of Dr David Carroll, Radiopaedia.org, rID: 64279. https://radiopaedia.org/cases/normal-chest-x-ray?lang=us Accessed 17 August 2020.

## Slide 13
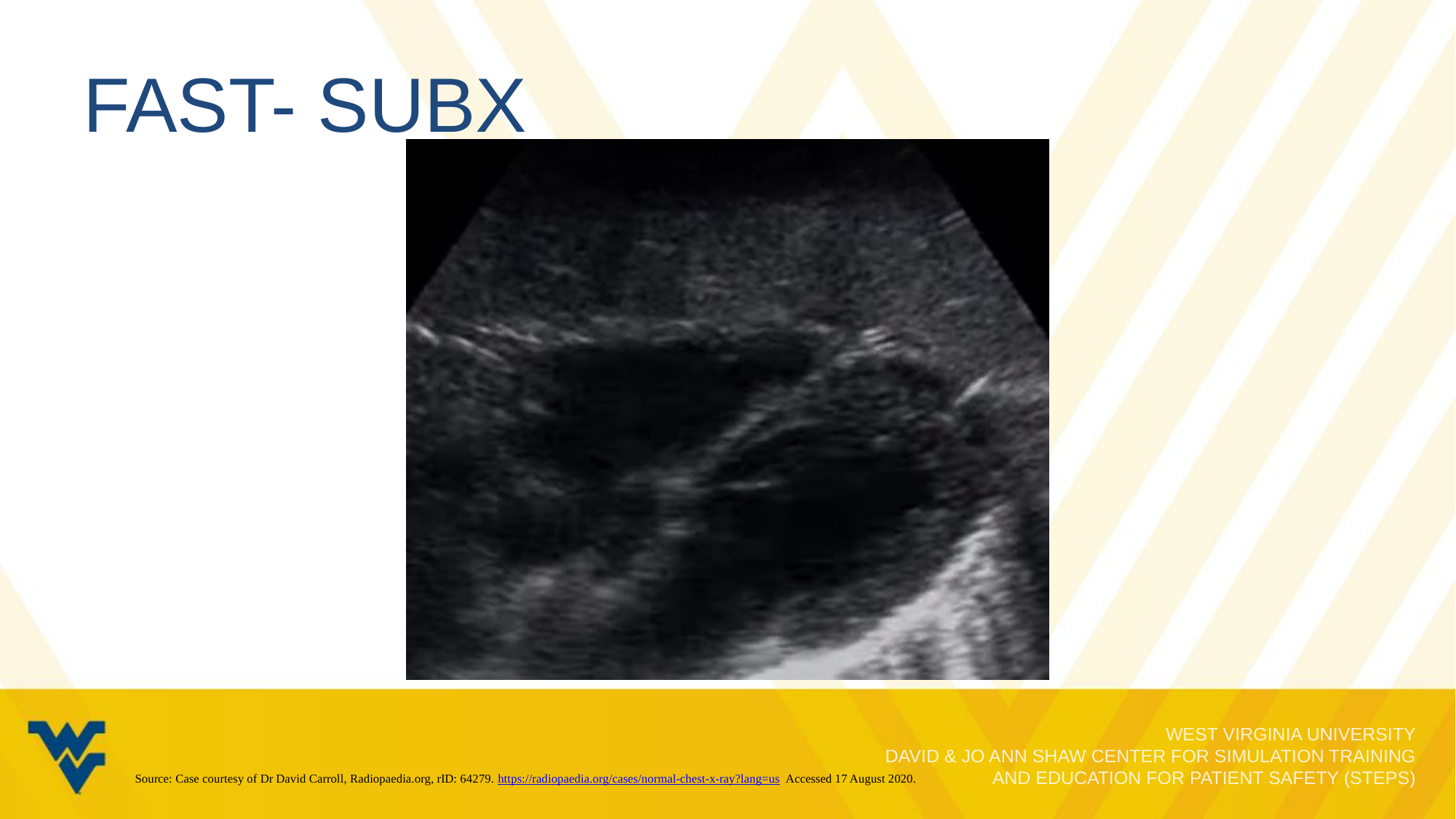

# FAST- subx
Source: Case courtesy of Dr David Carroll, Radiopaedia.org, rID: 64279. https://radiopaedia.org/cases/normal-chest-x-ray?lang=us Accessed 17 August 2020.
